# Anticonvulsant Effectiveness and Neurotoxicity Profile of 4-butyl-5-[(4-chloro-2-methylphenoxy)methyl]-2,4-dihydro-3*H*-1,2,4-triazole-3-thione (TPL-16) in Mice

**DOI:** 10.1007/s11064-020-03175-z

**Published:** 2020-11-18

**Authors:** Magdalena Drabik, Mariusz Głuszak, Paula Wróblewska-Łuczka, Zbigniew Plewa, Marek Jankiewicz, Justyna Kozińska, Magdalena Florek-Łuszczki, Tomasz Plech, Jarogniew J. Łuszczki

**Affiliations:** 1 Department of Pathophysiology, Medical University of Lublin, Jaczewskiego 8b, PL 20-090 Lublin, Poland; 2 Department of General, Oncological, and Minimally Invasive Surgery, 1 Military Clinical Hospital with the Outpatient Clinic in Lublin, Lublin, Poland; 3 Department and Clinic of Cardiology, Medical University of Lublin, Lublin, Poland; 4 Department and Clinic of Hematolooncology and Bone Marrow Transplantation, Medical University of Lublin, Lublin, Poland; 5 Department of Medical Anthropology, Institute of Rural Health, Lublin, Poland; 6 Department of Pharmacology, Medical University of Lublin, Lublin, Poland; 7 Isobolographic Analysis Laboratory, Institute of Rural Health, Lublin, Poland

**Keywords:** Antiepileptic drugs, Tonic-clonic seizures, Pharmacokinetic/pharmacodynamic interaction, Protective index, 1,2,4-triazole-3-thione

## Abstract

Protective (antiseizure) effects of 4-butyl-5-[(4-chloro-2-methylphenoxy)-methyl]-2,4-dihydro-3*H*-1,2,4-triazole-3-thione (TPL-16) and acute neurotoxic effects were determined in the tonic-clonic seizure model and rotarod test in mice. The interaction profile of four classic antiepileptic drugs (carbamazepine, phenobarbital, phenytoin and valproate) with TPL-16 was also determined in the tonic-clonic seizure model in mice. The protective effects of TPL-16 from tonic-clonic seizures (as ED_50_ values) and acute neurotoxic effects of TPL-16 (as TD_50_ values) were determined in 4 pretreatment times (15, 30, 60 and 120 min after its i.p. administration), in adult male albino Swiss mice. The interaction profile of TPL-16 with carbamazepine, phenobarbital, phenytoin and valproate in the tonic-clonic seizure model was determined with isobolographic analysis. Total concentrations of carbamazepine, phenobarbital, phenytoin and valproate were measured in the mouse brain homogenates. The candidate for novel antiepileptic drug (TPL-16) administered separately 15 min before experiments, has a beneficial profile with protective index (as ratio of TD_50_ and ED_50_ values) amounting to 5.58. The combination of TPL-16 with valproate produced synergistic interaction in the tonic-clonic seizure model in mice. The combinations of TPL-16 with carbamazepine, phenobarbital and phenytoin produced additive interaction in terms of protection from tonic-clonic seizures in mice. None of the total brain concentrations of classic AEDs were changed significantly after TPL-16 administration in mice. Synergistic interaction for TPL-16 with valproate and the additive interaction for TPL-16 with carbamazepine, phenobarbital and phenytoin in the tonic-clonic seizures in mice allows for recommending TPL-16 as the promising drug for further experimental and clinical testing.

## Introduction

Epilepsy is a neurological disease, which occurs in 6 per 1000 people worldwide with morbidity that differs according to ethnic origin [1]. In epilepsy patients both, unprovoked and spontaneous seizures caused by over-excitation of certain neurons, make this disease unpredictable [2]. Thereupon accurate treatment should be based on precise medical experience and investigation of brain activity by using EEG or MRI [2, 3]. Because of diversity among signs and symptoms of epilepsy seizures, choice of appropriate drug might be difficult [2]. In 70% of patients, monotherapy with current frontline antiepileptic drugs (AEDs) provides sufficient treatment of epileptic seizures [4, 5]. However, despite a variety of available AEDs there are still 30% of drug-resistant patients [3, 6]. Polytherapy is necessary and obligatory if physicians want to increase efficiency of the treatment or when two previous medications have failed [2, 4, 5].

Because of drug-resistant epilepsies or adverse effects of currently licensed AEDs, novel more efficient medications are needed [7, 8]. Recently, researches focused more on genetic aspects of the disease and its pathophysiology, finding some new structural and functional targets [9, 10]. Discovery of some specific targets and related compounds is one of the strategies, which should enable improvement in treatment of epilepsy. Other methods suggest changing a chemical structure of known AEDs or phenotypic screening of compounds with unknown mechanisms [7, 8]. In silico methods are the useful tools, which help to predict biological activity and structure of designed compounds [11]. Moreover, these tools are able to notice phenotypic and genotypic differences between AEDs in polytherapy [12]. Then leading compounds can be synthesized and can undergo preclinical investigations, so we can be sure about novel drugs reaching their target(s), especially, after crossing blood–brain barrier and acting in neurons—in the place of seizure initiation, amplification and/or propagation.

An example of compounds, which are now being intensively studied is 1,2,4-triazole-3-thione derivatives. Experimental studies have shown that they have various properties, including the anticonvulsant activity [13]. Additionally, the compounds interact with voltage-gated sodium channels [13–15]. Growing interest in 1,2,4-triazole-3-thione derivatives resulted in identifying therapeutic potential and permeability through the blood–brain barrier of various 1,2,4-triazole-3-thione derivatives [15–18]. Moreover, the most promising compounds, which presented a clear-cut effect in the maximal electroshock-induced seizure (MES) model, such as: 4-(4-bromophenyl)-5-(3-chlorophenyl)-2,4-dihydro-3*H*-1,2,4-triazol-3-thion (TP-4); 5-(3-chlorophenyl)-4-(4-methylphenyl)-2,4-dihydro-3*H*-1,2,4-triazol-3-thion (TP-10); 5-[(3-fluorophenyl)ethyl]-4-(n-hexyl)-2,4-dihydro-3*H*-1,2,4-triazole-3-thione (TPF-34); 5-(3-chlorobenzyl)-4-hexyl-2,4-dihydro-3*H*-1,2,4-triazole-3-thione (TP427), and 5-(3-chlorophenyl)-4-hexyl-2,4-dihydro-3*H*-1,2,4-triazole-3-thione (TP-315), have been investigated more precisely in terms of interaction and toxicity with classic AEDs [18–22].

One of the compounds, which has not been studied yet is 4-butyl-5-[(4-chloro-2-methylphenoxy)methyl]-2,4-dihydro-3H-1,2,4-triazole-3-thione (TPL-16). In this article we present in vivo experiments based on acute seizure model and the test assessing the drug’s toxicity potential in mice, along with evaluation of TPL-16 interactions with 4 classic AEDs. To evaluate the anticonvulsant properties of TPL-16, the mouse tonic-clonic seizure (MES) model was used. Of note, the MES test as a standard method in the research of new anticonvulsant compounds, enable us with reliable results that could be compared with other 1,2,4-triazole-3-thione derivatives [13].

This work aimed to evaluate anticonvulsant effectiveness and tolerability of TPL-16. Its possible neurotoxic influence on motor coordination in animals was assessed in the rotarod test. Consistent with The Epilepsy Therapy Screening Program (ETSP), protective indices were calculated based on the anticonvulsant and motor impartment assessments at 4 different pretreatment times, as recommended elsewhere [23]. Another purpose of this study was to assess the interaction profile of TPL-16 with four classic AEDs (including, carbamazepine, phenobarbital, phenytoin, and valproate) using the mouse MES model by means of isobolographic analysis. Interaction profile was also established with respect to pharmacokinetic properties of the examined compound TPL-16 and measured by total brain concentrations of classic AEDs. Reason for choosing these four classic AEDs was related to the fact that they are commonly used in both preclinical practice and clinical settings.

## Materials and Methods

### Animals

All experiments were conducted on adult (8–9 week old) male albino Swiss mice weighing 20–26 g. After a week of acclimatization to laboratory conditions, the animals were randomly divided into experimental groups of 8 mice per each. The experiments were carried out between 8.00 and 15.00 to avoid disruption of circadian rhythm, and each animal was used only once in the tonic-clonic seizure model. All experimental procedures described below were carried out in accordance with the standards of care and use of laboratory animals and have been approved by the Local Ethics Committee (approval no. 88/2018 from 2nd July, 2018).

### Drugs

**Fig. 1:**
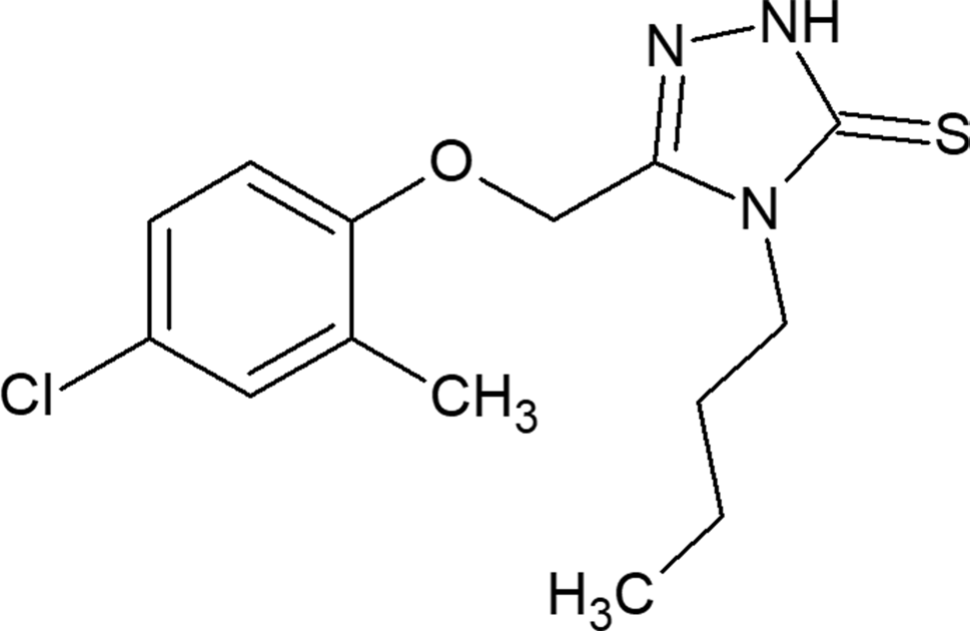
Chemical structure of 4-butyl-5-[(4-chloro-2-methylphenoxy)methyl]-2,4-dihydro-3*H*-1,2,4-triazole-3-thione (TPL-16)

4-butyl-5-[(4-chloro-2-methylphenoxy)methyl]-2,4-dihydro-3*H*-1,2,4-triazole-3-thione (TPL-16—Fig. 1) was synthesized by prof. T. Plech and evaluated in this study. TPL-16 was dissolved in a 1% solution of Tween 80 *v*/v (Sigma-Aldrich). The mice received intraperitoneally (i.p.) various increasing doses of TPL-16 at the appropriate concentration and volume of 5 ml/kg body weight at four different pretreatment times (i.e., 15, 30, 60 and 120 min). Carbamazepine (Polpharma, Starogard Gdanski, Poland), phenobarbital (Polfa, Krakow, Poland), phenytoin and valproate (both from Sigma-Aldrich, Poznan, Poland) were injected i.p. as follows: PHT—120 min, PB—60 min, CBZ and VPA—30 min prior to the electrically-evoked tonic-clonic seizures, behavioral tests and measurement of total brain AED concentrations, as reported elsewhere [20, 22, 24]. Of note, these pretreatment time points for classic AEDs were selected based on their peak of anticonvulsant activity in experimental animals [25].

### Maximal Electroshock-Induced Seizure (MES) Test in Mice

The MES test was accepted by ETSP as a standard method of evoking tonic-clonic seizures in rodents [23]. By the Rodent Shocker generator (Hugo Sachs Elektronik, Freiburg, Germany) an alternating current (50 Hz; 500 V; 25 mA) of 0.2 s duration via ear-clip electrodes was delivered to the animals. After randomized selection of the laboratory animals into groups consisting of 8 animals, the mice received increasing doses of TPL-16. To evaluate the antiseizure effect of TPL-16, the MES test was conducted in four different pretreatment times (i.e., 15, 30, 60, 120 min). According to the log-probit method, the median effective dose (ED_50_) of TPL-16 (in mg/kg) reflects rodents’ protection from tonic-clonic seizures in the MES test [26]. Total number of mice used in this screening was 96. To assess the anticonvulsant properties of classic AEDs (carbamazepine, phenobarbital, phenytoin, valproate), the drugs were administrated i.p. separately, according to their pretreatment times. Their ED_50_ values, according to the log-probit method, were estimated based on the MES test results [26]. To conduct this screening, additional 96 mice were used. During the isobolographic analysis, TPL-16 was administrated i.p. in combination with each of the studied classic AEDs (all drugs at increasing doses in a fixed drug dose ratio of 1:1). Subsequently, median effective dose (ED_50 mix_) of TPL-16 combined with a proper classic AED was established based on the MES test results, in respect to the log-probit method [26]. To conduct isobolographic analysis of interaction in the MES test 104 mice were used. Summarizing, to perform anticonvulsant screening tests in the mouse MES model were used totally 296 mice. After evaluating the MES-induced seizure activity in male albino Swiss mice and finishing the experiments, the animals immediately underwent euthanasia by means of carbon dioxide (CO_2_), as recommended elsewhere [27].

### Rotarod Test in Mice

To evaluate the potential of impaired motor activity in rodents after receiving TPL-16, rotarod test was conducted. The rotarod apparatus (Ugo Basile, Comerio Varese, Italy) allows for determining whether the animals that received the respective doses of TPL-16 had impaired motor coordination, as described elsewhere [21]. In this test, the mice have to remain in equilibrium for 120 s on the rotating cylinders with a constant speed of 6 rpm. In this study 96 mice were divided into the groups comprising 8 animals and received increasing doses of TPL-16. Then the mice were subjected to the examination of their motor coordination at 4 different pretreatment times (15, 30, 60 and 120 min). Determination of median toxic doses (TD_50_) was based on recording the number of rodents in each group which failed the rotarod test. The TD_50_ reflects acute neurotoxicity of the investigated compound, which affects balance and motor coordination in animals. The determination of TD_50_ values in the rotarod test was performed with log-probit method [26].

### Isobolographic Analysis of Data

Isobolographic analysis is commonly used in evaluating interactions between investigated compounds [28, 29]. It compares median additive doses (ED_50 add_), theoretically calculated from the particular ED_50_ values of the studied AEDs and TPL-16, to the experimentally determined median effective doses (ED_50 mix_) of TPL-16 and classic AEDs, which reflect the protective (anticonvulsant) effect in 50% of rodents subjected to the MES test. Interactions for the combination of TPL-16 with each of the studied classic AEDs were examined isobolographically. To properly classify interaction for two-drug combinations, the interaction index (Ω) value was calculated by dividing the respective ED_50 mix_ value and ED_50 add_ value [30, 31].

### Potential Side Effects of TPL-16 in Combination with Classic Antiepileptic Drugs in Mice

To evaluate potential side effects produced by the combination of TPL-16 with classic AEDs (carbamazepine, phenobarbital, phenytoin, valproate) two behavioral (the chimney and grip-strength) tests were conducted in mice.

#### Chimney Test in Mice

To investigate the effects of the AEDs combined with TPL-16 on motor coordination of animals, the chimney test was used [32]. To carry out the experiment, a cylinder (3 cm in diameter and 30 cm in length), whose inner wall has a rough surface, was used. Mice were individually introduced into a horizontally positioned cylinder. When the animal passed to the other end, the cylinder was placed vertically. The animal’s task was to exit it backwards up during 60 s. The impaired motor coordination was found in mice that failed to correctly perform the test. For this procedure 48 mice were used.

#### Grip-Strength Test in Mice

A grip-strength test was used to evaluate the side effects of AEDs co-administered with TPL-16 on skeletal muscle strength, as recommended elsewhere [33]. For this purpose, a 8 × 8 cm metal mesh was used, which the mouse is intended to grasp, and an apparatus consisting of a force transducer that records the strength of the animal’s forelimb muscles. The mouse, held by the tail, was lowered onto a metal mesh to catch it with its front paws. Then the animal was gently pulled backwards in a horizontal motion until it let go of the mesh. The maximal force registered by the device is the moment just before the loss of adhesion. The result is the average of three measurements for each mouse. The mean of the maximal strength of the animals was expressed in millinewtons per gram of the body weight (mN/g). For this procedure 48 mice were used.

### Total Brain Antiepileptic Drug Concentration Measurements

Total brain AED concentrations were measured using fluorescence polarization immunoassay in mice. Prior to the decapitation and brain samples preparation, the animals received classic AEDs alone and in combination with TPL-16, according to the ED_50 mix_ values established during the MES test, as described elsewhere [20, 22, 24]. Concentration values of detected AEDs were presented in μg/g wet brain tissue of 8 separately prepared brains (as means ± SEM). For this procedure 64 mice were used.

### Statistical Analysis

Anticonvulsant effectiveness and tolerability screening tests of TPL-16 with experimentally-derived ED_50_ and TD_50_ values (± SEM) at 4 different pretreatment times (15, 30, 60 and 120 min) were statistically analyzed with one-way ANOVA followed by a post test for linear trend. The isobolographic analysis, included evaluation of TPL-16 correlation with classic AEDs (carbamazepine, phenobarbital, phenytoin, valproate) according to previously described dose-response log-probit method [26]. The unpaired Student t-test was applied in comparing the respective ED_50 mix_ and ED_50 add_ values of combined compounds and in statistical analysis of total brain concentrations of the investigated AEDs. The results from the chimney test were analyzed by the Fisher’s exact probability test. The results from the grip-strength test were statistically analyzed by one-way ANOVA. Statistical significance was complied with the *p* < 0.05. All statistical calculations were performed using GraphPad Prism (version 7.0 for Windows), whereas the power analysis by means of G*Power software (version 3.1.9.7 for Windows).

## Results

### Protective (Antiseizure) Effects of TPL-16 from MES-Induced Seizures and Acute Neurotoxic Effects of TPL-16 in the Rotarod Test in Mice

**Fig. 2:**
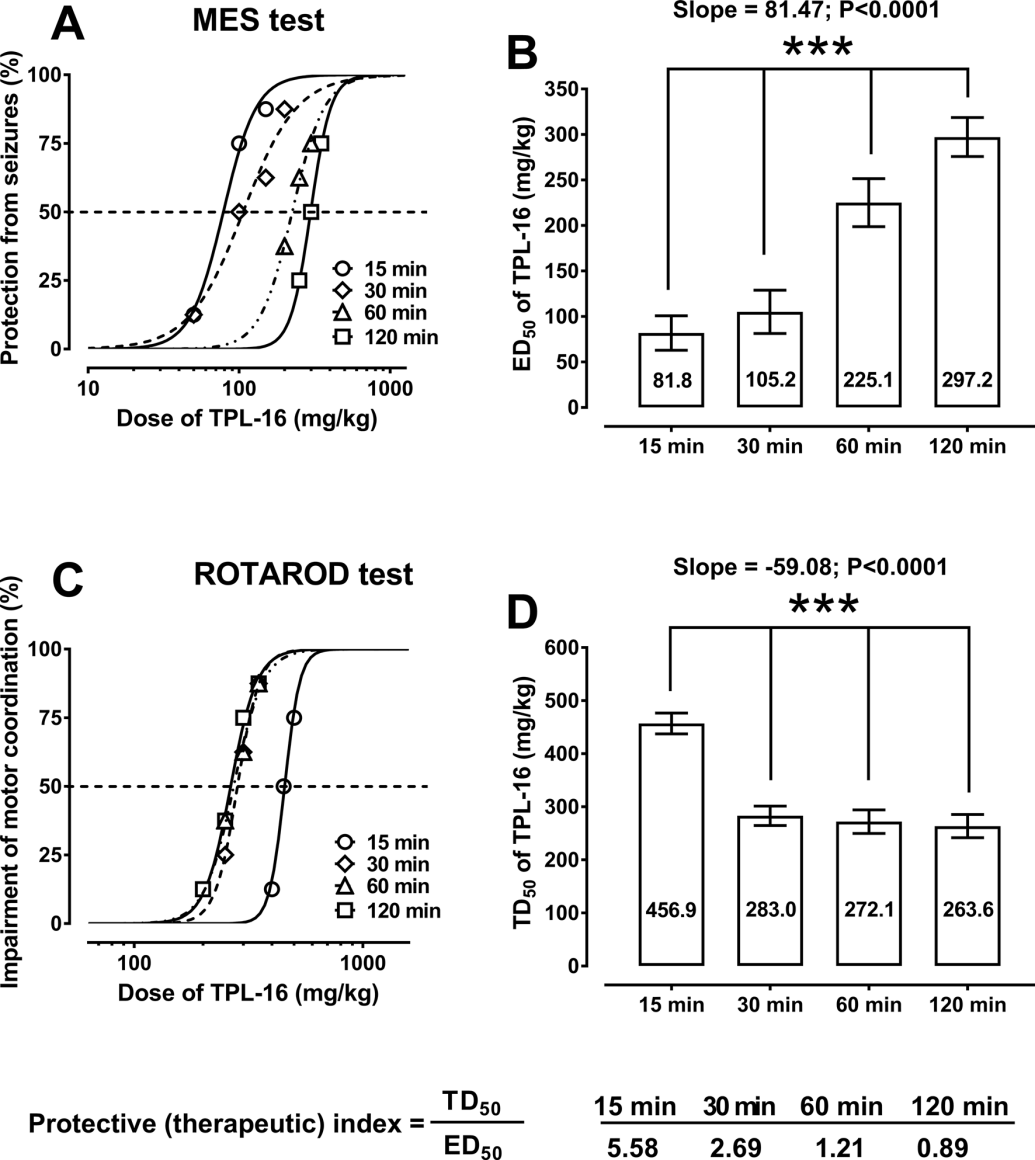
(**a**–**d**) Protective (antiseizure) and acute neurotoxic effects of TPL-16 in the maximal electroshock (MES)-induced seizure model and rotarod test in mice. The graph (**a**) shows 4 dose-effect curves for TPL-16 administered singly i.p. at 4 different pretreatment times (i.e., 15, 30, 60 and 120 min) before the MES test in mice. Each point illustrates the dose of TPL-16 injected to a group of 8 mice and the protective effect of TPL-16 from tonic-clonic seizures in the MES test. The graph (**b**) presents 4 columns as ED_50_ values (in mg/kg) for TPL-16 at 4 different pretreatment times in the tonic-clonic seizure model in mice. The ED_50_ values are represented as columns with error bars as their SEM. The ED_50_ values for TPL-16 at 4 different pretreatment times (i.e., 15, 30, 60 and 120 min) were statistically analyzed with linear trend that showed significance (***p < 0.0001). The graph (**c**) shows 4 dose-effect curves for TPL-16 administered singly i.p. at 4 different times (i.e., 15, 30, 60 and 120 min) before the rotarod test in mice. Each point illustrates, on the X axis, the dose of TPL-16 injected to a group of 8 mice, and on the Y axis, the toxic effect of TPL-16 manifested in the form of impaired motor coordination in mice challenged with the rotarod test. The graph (**d**) shows 4 columns as TD_50_ values ​​(in mg/kg) for TPL-16 at 4 different pretreatment times in the rotarod test in mice. The TD_50_ values ​​are represented as columns with error bars as their SEM. The TD_50_ values for TPL-16 at 4 different pretreatment times (i.e., 15, 30, 60 and 120 min) were statistically analyzed with linear trend that showed significance (***p < 0.0001). Both, ED_50_ and TD_50_ values were calculated from the log-probit method. Protective index was calculated as a ratio of TD_50_ and ED_50_ values in various 4 pretreatment times

Single systemic (i.p.) administration of TPL-16 at 4 pretreatment times (i.e., 15, 30, 60 and 120 min) produced, in a dose-dependent manner, the anticonvulsant effects in the tonic-clonic seizure model in mice (Fig. 2a). The ED_50_ values for TPL-16 linearly increased from 81.8 mg/kg to 297.2 mg/kg (Fig. 2b). One-way ANOVA with post-hoc analysis of a trend among the ED_50_ values revealed significance (F [1;68] = 31.38; *p* < 0.0001).

Similarly, TPL-16 injected i.p. at 4 pretreatment times (i.e., 15, 30, 60 and 120 min) exerted, in a dose-dependent manner, the acute neurotoxic effects in mice challenged with the rotarod test (Fig. 2c). Acute side effects manifested as impairment of motor coordination in mice and the TD_50_ values for TPL-16 linearly decreased from 456.9 mg/kg to 263.6 mg/kg (Fig. 2d). One-way ANOVA with post-hoc analysis of a trend among the TD_50_ values confirmed significance (F [1;60] = 41.13; *p* < 0.0001). The protective (therapeutic) index values for TPL-16 ranged from 5.58 to 0.89, at 4 respective pretreatment times (Fig. 2D).

Total number of animals used to determine 4 ED_50_ values in the MES test and 4 TD_50_ values ​​in the rotarod test was identical amounting to 104 mice (13 groups of 8 mice) in each test. Thus, the power analysis for 104 mice with a probability of making a type I error (alpha) of 0.05 and an effect size of 0.4, for 4 different ED_50_ or TD_50_ values ​​is 0.93 (post-hoc F-test ANOVA). In turn, the minimal number of animals (sample size) necessary to obtain the power of experiment 0.9, with a probability of making a type I error (alpha) equals to 0.05 and an effect size equals to 0.4, for 4 different ED_50_ or TD_50_ values ​​is 96 mice (a priori F-test ANOVA).

### Protective (Anticonvulsant) Effects of Four Classic AEDs and TPL-16 from Tonic-Clonic Seizures in Mice

**Fig. 3:**
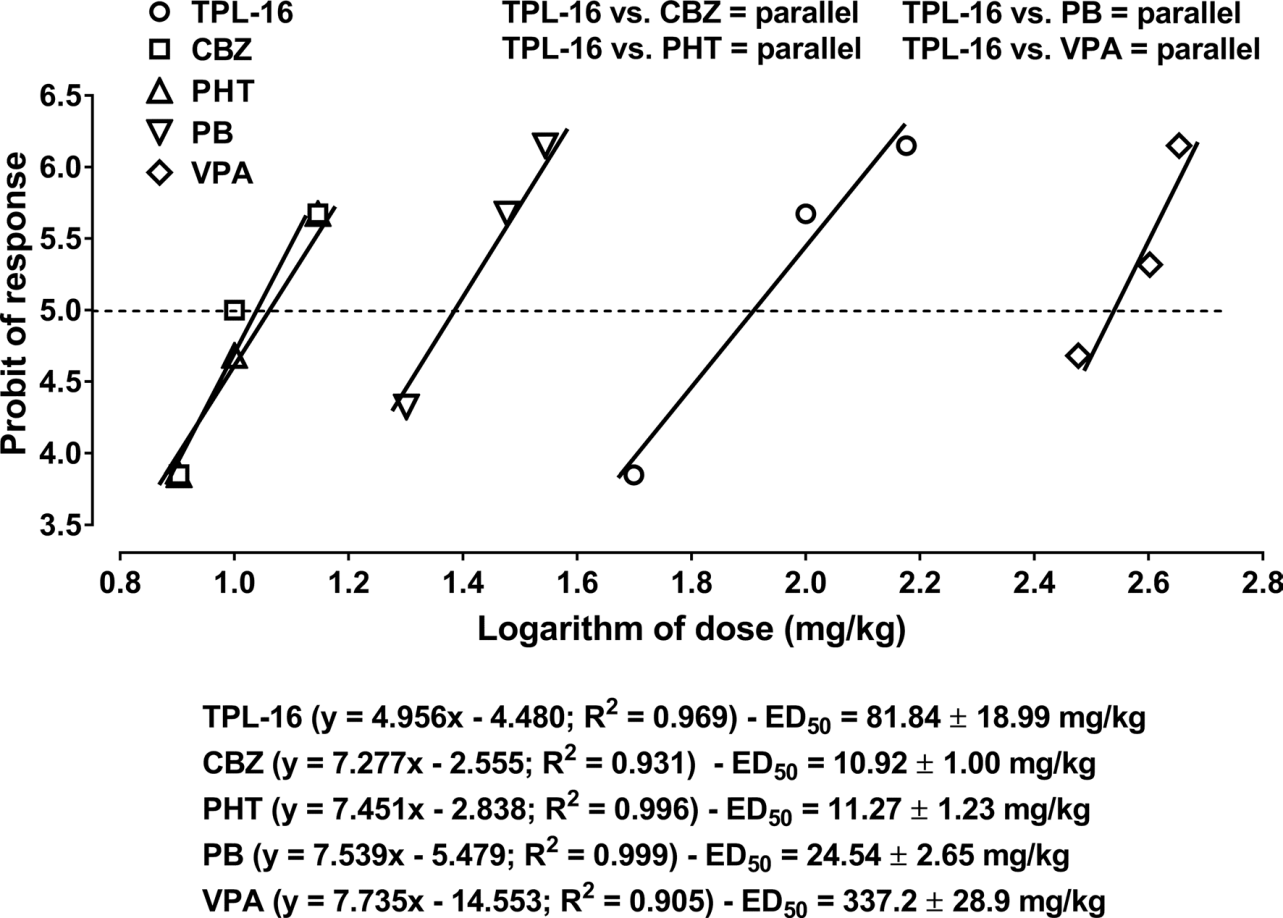
Protective (antiseizure) effects of four classic AEDs and TPL-16 in the maximal electroshock (MES)-induced seizure test in mice. The experimentally-derived ED_50_ values (± SEM) for 4 classic AEDs and TPL-16 were determined according to the log-probit method, which allowed performing the test of parallelism between dose-response effect line of TPL-16 and those of the classic AEDs. Test of parallelism revealed that the respective lines are collateral. For each drug the respective log-probit equation along with the ED_50_ value is presented below the graph. On the Y axis, at the height of the 5th probit, a straight dashed line parallel to the X axis reflects the likely ED_50_ values ​​for CBZ, PHT, PB, VPA and TPL-16

Single systemic (i.p.) administration of carbamazepine (CBZ), phenobarbital (PB), phenytoin (PHT) and valproate (VPA) produced, in a dose-dependent manner, the anticonvulsant effects in the mouse tonic-clonic seizure model (Fig. 3). Comparison of dose-response effect lines for classic AEDs with TPL-16 with test of parallelism confirmed that all classic AEDs had their dose-response effect lines collateral to that of TPL-16 (Fig. 3).

### Isobolographic Interactions Between TPL-16 and Classic Antiepileptic Drugs in the Tonic-Clonic Seizure Model in Mice

**Fig. 4:**
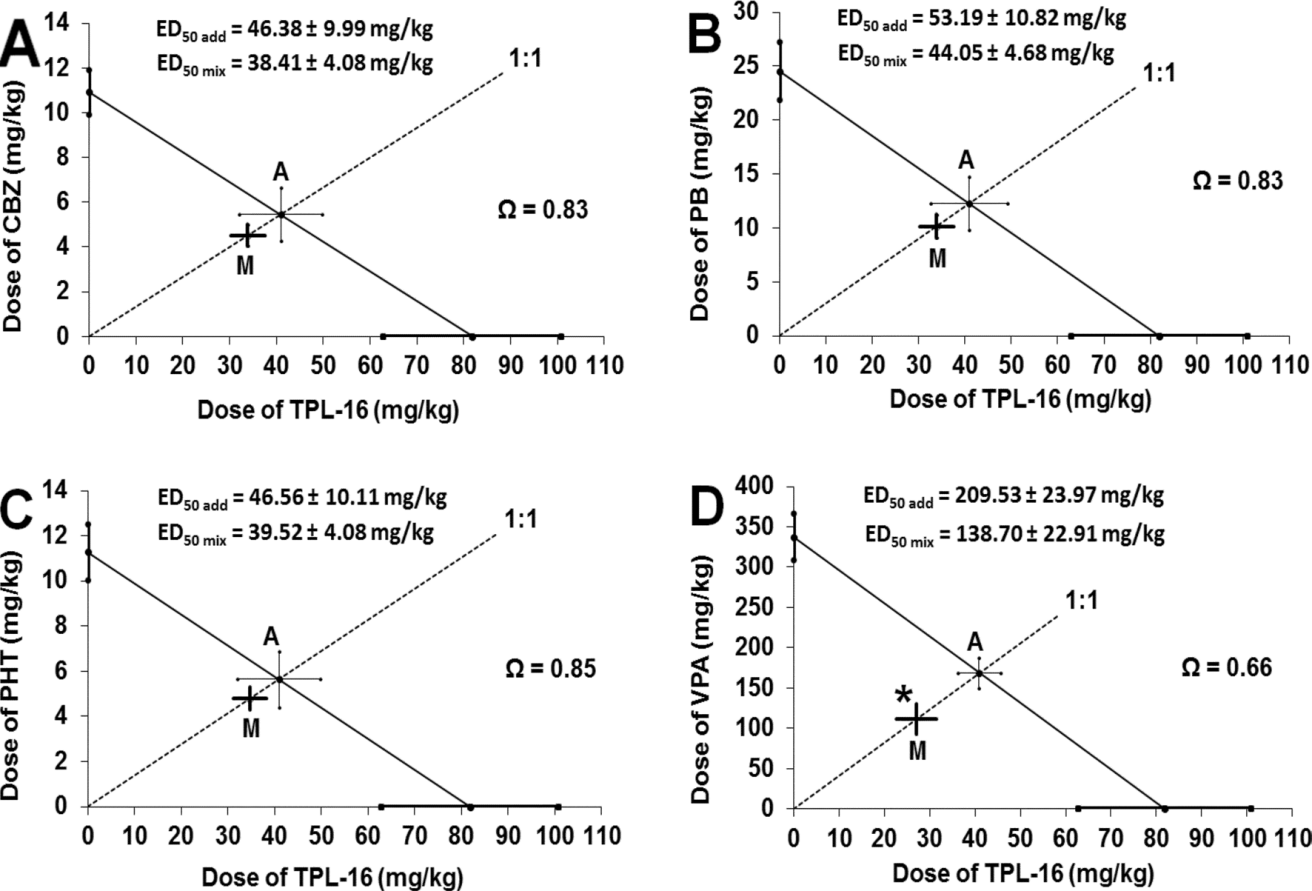
(**a**–**d**) Isobolograms illustrating additive interactions between TPL-16 and carbamazepine (**a**), phenobarbital (**b**), phenytoin (**c**), and synergistic interaction between TPL-16 with valproate (**d**) in the tonic-clonic seizure model in mice. The ED_50_ (± S.E.M.) values for TPL-16 and CBZ (**a**), PB (**b**), PHT (**c**) and VPA (**d**), given separately, are placed in the Cartesian coordinate system. The diagonal line connecting the ED_50_ values ​​for TPL-16 and classic AEDs, given separately, is the line of additivity. The dashed line starting from origin of the system and intersecting the additivity line illustrates a constant dose ratio of 1:1 for the mixture of both drugs, i.e., TPL-16 and CBZ (**a**), PB (**b**), PHT (**c**) and VPA (**d**). The ED_50 mix_ values (± SEM) for the combination of TPL-16 and classic AEDs are placed on the dashed lines, which correspond to the point M on each graph. The ED_50 add_ values (± SEM) for the combination of TPL-16 and classic AEDs are placed on the diagonal lines which intersected the dashed lines and reflect the point A on each graph. The unpaired Student’s t-test with Welch correction showed no significant differences between the ED_50 mix_ and ED_50 add_ values for the combination of TPL-16 with CBZ, PB and PHT, and thus, the interaction between the drugs shows additivity. Moreover, the interaction indices (Ω), characterizing the strength of the interaction between TPL-16 and CBZ, PB, PHT, suggest isobolographic additivity in terms of protection from tonic-clonic seizures in mice. However, a significant difference (*p < 0.05) was shown between the ED_50 mix_ and ED_50 add_ values for TPL-16 combined with VPA, whereby the interaction between those compounds shows supra-additivity (synergy) in the mouse tonic-clonic seizure model. In this case, the interaction index (Ω) for the combination of TPL-16 with VPA, suggests isobolographic supra-additivity with respect to the protection from tonic-clonic seizures in mice

The combination of TPL-16 with CBZ, PB and PHT (in a fixed dose ratio of 1:1) protected the animals from tonic-clonic seizures in the MES test in mice in an additive manner because the experimentally determined ED_50 mix_ values for these combinations did not differ significantly (according to Student’s t-test with Welch correction) from the corresponding ED_50 add_ values (Fig. 4a–c). Interaction indices (Ω) for these combinations ranged between 0.85 and 0.83 (Fig. 4a–c). In contrast, the combination of TPL-16 with valproate (VPA) at a constant dose ratio of 1:1 in the MES test in mice synergistically protected the mice from tonic-clonic seizures because the experimentally determined ED_50 mix_ value for the combination significantly differed (according to Student’s t-test with Welch correction; *p* < 0.05) from the corresponding ED_50 add_ value (Fig. 4d). The interaction index (Ω) for this combination amounted to 0.66, indicating synergy in the antiseizure action between the tested drugs (Fig. 4d).

### Impact of TPL-16 on Total Brain Antiepileptic Drug Concentrations

**Fig. 5:**
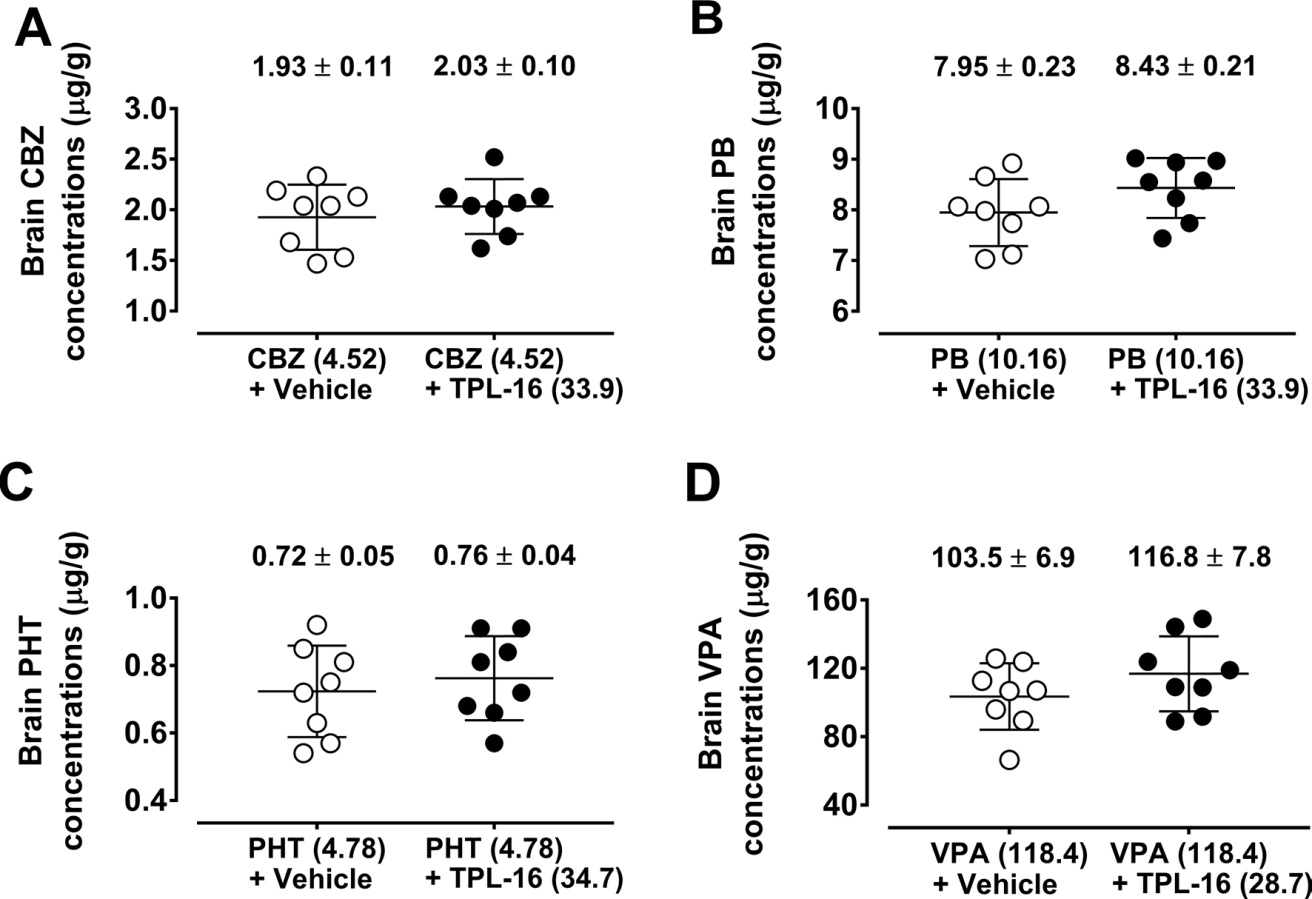
(**a**–**d**) Impact of TPL-16 on total brain concentrations of the classic AEDs in mice. Total brain concentrations of classic AEDs as means ± SEM (*n* = 8 mice per group) are expressed in μg/g of wet brain tissue. No statistical significance between the means of classic AEDs was observed with unpaired Student’s t-test. CBZ—carbamazepine, PB—phenobarbital, PHT—phenytoin, VPA—valproate

The unpaired Student’s t-test revealed that TPL-16 did not significantly alter total brain concentrations of all classic AEDs studied in experimental animals (Fig. 5a–d).

### Potential Side Effects of TPL-16 in Combination with Classic Antiepileptic Drugs in Mice

The combinations of TPL-16 with CBZ, PB, PHT and VPA, administered in doses reflecting their constant dose ratio of 1:1 derived from the mouse tonic-clonic seizure test, showed no impairment in motor coordination in mice challenged with the chimney test (Table 1). Similarly, TPL-16 in combination with CBZ, PB, PHT and VPA, administered in doses reflecting their constant dose ratio of 1:1 derived from the mouse tonic-clonic seizure test, did not show any significant impairment in skeletal muscular strength in mice subjected to the grip-strength test (Table 1).

**Table 1:** Impact of TPL-16 in combinations with classic AEDs on motor coordination and skeletal muscular strength in mice challenged with the chimney and grip-strength tests

Drugs in combination (mg/kg)	Motor coordination impairment (%)	Skeletal muscular strength (mN/g)
Vehicle + vehicle	0	36.66 ± 2.47
CBZ (4.52) + TPL-16 (33.9)	0	35.87 ± 2.80
PB (10.16) + TPL-16 (33.9)	0	36.83 ± 2.28
PHT (4.78) + TPL-16 (34.7)	0	37.80 ± 2.38
VPA (118.4) + TPL-16 (28.7)	0	36.33 ± 2.59

Results are presented as: (1) percent impairment of motor coordination in mice receiving the combination of TPL-16 with each of the classic AEDs studied as compared to the control group (vehicle + vehicle-treated animals), (2) mean strength (± SEM) of the skeletal muscles of the forelimbs in mice (in millinewtons per gram body of the test animals), receiving TPL-16 in combination with CBZ, PB, PHT and VPA, or vehicle + vehicle (control group). Each experimental group consisted of 8 mice. The Fisher’s exact probability test revealed no significant difference in motor coordination in mice. The one-way ANOVA showed no statistical significance between skeletal muscular strength in mice. CBZ—carbamazepine, PB—phenobarbital, PHT—phenytoin, VPA—valproate

## Discussion

The investigated substance TPL-16, after a single administration showed a protective effect against tonic-clonic seizures in the MES test in mice at 4 established intervals when administrated systemically (i.p.) i.e., 15, 30, 60 and 120 min before the MES test. The mentioned 4 time intervals are the standard times for in vivo testing when screening for substances with anticonvulsant potential [34]. It should be emphasized that screening tests (such as the MES test in mice) carried out at 4 basic times (15, 30, 60 and 120 min) assess the potential for anticonvulsant activity of the examined substances. The first anticonvulsive screening indicates whether the investigated compound possesses any anticonvulsant activity by protecting mice against tonic-clonic seizures in the MES test. In addition to 1,2,4-triazole-3-thiones, this anticonvulsive screening has recently been carried out for many other chemical derivatives, including 1,3,4-thiadiazoles [35], p-isopropoxyphenylpyrrolidine-2,5-diones [36] or picolinic acid benzylamides [37]. The results of such screening allow for preferential selection of substances that produce anticonvulsant activity in the MES test in mice and might be subjected to further preclinical tests to determine their pharmacological profile.

In the next stage of the study, the effectiveness of TPL-16 in inhibiting tonic-clonic convulsions in mice was assessed by determining its ED_50_ values at 4 different pretreatment times. The MES test showed that the longer the time interval between administration of the investigated substance TPL-16 and the induction of convulsive activity by electric current, the weaker its protective effect against electrically-induced convulsions, due to the highest ED_50_ value for TPL-16 after 120 min from its administration. A statistically significant linear trend of increase in ED_50_ values for TPL-16 over time was also observed to protect against seizures in mice. It is worth noting that, for the first time, in this study statistical analysis of the ED_50_ values over time was performed, using one-way ANOVA with post-hoc linear trend to emphasize the importance of the phenomenon of loss of anticonvulsant activity of the tested substance TPL-16 in the MES test in mice. The linearity of the ED_50_ increase within 120 min suggests that TPL-16 is probably degraded in vivo into inactive or less active metabolites, which reduces its anticonvulsant effect. Also a first-pass effect through the liver should be taken into account, i.e. the deactivation of the drug by microsomal liver enzymes [38]. It is noteworthy that the peak of anticonvulsant activity of various 1,2,4-triazole-3-thione derivatives ranges from 15 min, e.g. for TPL-16 and TPF-34 [21], up to 120 min e.g. for 5-(4-chlorophenyl)-4-(2,4-difluorophenyl)-2,4-dihydro-3H-1,2,4-triazole-3-thione and 4-(4-bromophenyl)-5-(4-chlorophenyl)-2,4-dihydro-3H-1,2,4-triazole-3-thione [14, 16].

Based on the calculated ED_50_ values, it was considered that 15 min after the i.p. administration of the compound was the peak of the protective effect of TPL-16 in mice. By simultaneously using 4 standard times to perform in vivo screening studies, the potential of TPL-16 toxicity in a mouse rotarod test was also assessed. When determining the TD_50_ values for TPL-16, it was shown that the longer the time interval between administration of TPL-16 and the performance of the rotarod test, the more severely impaired motor coordination of animals was in this study. In the case of the rotarod test, there was also a statistically significant linear trend of the decrease in TD_50_ for TPL-16 over time. Of course, the linearity of the TD_50_ decrease in 120 min suggests that TPL-16 is degraded into toxic metabolites in vivo in mice, which reduce motor coordination in mice. The rodent rotarod test is a standard test used in screening studies on compounds with anticonvulsant potential [23, 39, 40]. It is noteworthy that the peak of 1,2,4-triazole-3-thione toxicity varies from 15 min, e.g., for 5-(3-chlorophenyl)-4-(4-fluorophenyl)-2,4-dihydro-3H-1,2,4-triazole-3-thione, 4-(4-bromophenyl)-5-(3-chlorophenyl)-2,4-dihydro-3H-1,2,4-triazole-3-thione, 5-(3-chlorophenyl)-4-ethyl-2,4-dihydro-3H-1,2,4-triazol-3-thione, 4-butyl-5-(3-chlorophenyl)-2,4-dihydro-3H-1,2,4-triazole-3-thione [14, 16, 41], up to 120 min e.g. for 4-(2-bromophenyl)-5-(4-chlorophenyl)-2,4-dihydro-3H-1,2,4-triazole-3-thione, 5-(3-chlorophenyl)-4-hexyl-2,4-dihydro-3H-1,2,4-triazole-3-thione and TPL-16 [14, 16, 41]. In order to reliably perform the analysis of significance between individual ED_50_ and TD_50_ values ​​for TPL-16 administered alone in 4 pretreatment times, the power, effect size and sample size were calculated. All these components belong to intermediate steps that allow confirming in preclinical studies that the results obtained are not a coincidence and, at the same time, exclude the influence of interfering factors that could lead to the adoption of a false null hypothesis [42, 43]. It seems very important to determine the sample size before conducting the experiment, in order to ethically use the smallest possible number of animals for experiments, which are required by the ARRIVE procedures and guidelines [44] and, at the same time, from the statistical viewpoint to use the maximal number of animals so as to the sample was representative [45]. The power analysis for TPL-16 from the MES and rotarod tests gave a clear answer that the experiments were reliably carried out and the obtained results were not incidental.

Using the determined TD_50_ and ED_50_ values for TPL-16 administered i.p. at 4 standard intervals, the therapeutic index for TPL-16 was calculated as the quotient of TD_50_ and ED_50_ values determined previously at each time point. The highest value of the therapeutic index for TPL-16 was reached after 15 min from its i.p. administration and it was 5.58, and the lowest—after 120 min from administration and it was 0.89. Thus, a time of 15 min from administration of the analyzed compound to conducting convulsive and behavioral tests in mice was chosen as the optimal time for further testing and evaluation of the interaction profile of TPL-16 with 4 classic AEDs.

It should be emphasized that substances with anticonvulsant potential are assessed in in vivo screening tests and are subject to initial verification whether they are suitable for further investigation. The main criterion for selecting compounds for further in vivo studies is their therapeutic index, whose value should be greater than 5 for substances with anticonvulsant activity [34, 39, 40]. It is assumed that substances with a therapeutic (protective) index less than 5 should not participate in further experimental studies in epilepsy animal models. However, taking into account the fact that currently the most effective AED—valproate (used in adults and children with different types of epileptic seizures) has a therapeutic index in the MES test of less than 2, so if valproate were subject to the procedure of searching for drugs with antiseizure potential, then could be eliminated from future research because it does not meet the criterion of therapeutic index greater than 5. Therefore, in order not to eliminate some clinically efficacious drugs, some authors suggest that new substances with anticonvulsant properties subjected to screening tests for which the therapeutic index is less than 5 and greater than 2 also should be included in further preclinical studies [23]. At present, in the therapy of particularly drug-resistant epilepsy, third-generation AEDs with high protective indices exceeding 10 and more are used [46]. An example of a drug with a very high therapeutic index is levetiracetam, for which the therapeutic index in preclinical studies is above 100 [47]. It should also be noted that the tested substances derived from 1,2,4-triazole-3-thiones, such as: TPL-16, TPF-34, TP-315 are characterized by high therapeutic indices exceeding 5 [18, 21]. From among all tested 1,2,4-triazole-3-thione derivatives, the highest value of therapeutic index was achieved by: 4-butyl-5-(3-chlorophenyl)-2,4-dihydro-3H-1,2,4-triazol-3-thione—the therapeutic index equals to 10.3 [41] and 5-(3-chlorobenzyl)-4-hexyl-2,4-dihydro-3H-1,2,4-triazole-3-thione—the therapeutic index equals to 13.9 [16], 15 min after their i.p. administration. On the other hand, we are fully aware of the fact that a “standard” for further development of novel compounds, which require a high protective index is useful, but may limit exploration of novel mechanisms of the antiseizure action or discovery of novel compounds effective in distinct epilepsy populations.

Having performed a preliminary assessment of the anticonvulsant and toxic profile of TPL-16, the anticonvulsant effect of the combination of TPL-16 with 4 classic AEDs, such as carbamazepine (administered for 30 min), phenobarbital (administered for 60 min), phenytoin (administered for 120 min) and valproate (administered for 30 min) in the MES test in mice was assessed. Using the type I isobolographic analysis for parallel dose-effect lines, the doses of the listed drugs with TPL-16 were determined to be tested together in the mixture in a constant dose ratio of 1:1.

Noteworthy, the ED_50_ values ​​for classic AEDs (carbamazepine, phenobarbital, phenytoin and valproate) when used alone in the MES test in mice, were determined previously while assessing the nature of classic AEDs interactions with TPF-34 [21]. The use of the same ED_50_ values ​​for classic AEDs in two experimental studies was associated with obtaining one common ethical consent for conducting experiments for 2 substances TPL-16 and TPF-34 in the MES test in mice. According to the ARRIVE guidelines, the use of common ED_50_ values ​​for classic AEDs allowed to reduce the number of mice necessary for experiments according to the 3R rule [44, 48].

The log-probit method revealed that TPL-16 and classic AEDs had their dose-effect relationship lines collateral. In the next stage of the study, the isobolographic analysis of interaction between TPL-16 and classic AEDs in a constant dose ratio of 1:1 showed additive interactions in combination with carbamazepine, phenobarbital, phenytoin and supra-additive (synergistic) interaction in combination with valproate in the MES test in mice. The resulting interactions of additivity and synergy for TPL-16 with classic AEDs in the MES test in mice are very similar to those obtained earlier. For instance, TPF-34 showed additive interactions in the MES test in mice when co-administered with carbamazepine, phenytoin, phenobarbital and valproate [21]. In turn, TP-427 showed additive interactions in combination with carbamazepine, phenytoin and phenobarbital and synergistic interaction in combination with valproate [22]. Although there is a similarity in the results obtained for TPL-16 and TP-427 in their induction of similar interactions with classic AEDs in the MES test in mice, important differences (due to the methodology of both studies) should be emphasized. The interaction profile of TPL-16 was assessed according to the classic isobolographic analysis method, for which a fixed proportion of doses of both compounds included in the mixture was selected (1:1), while the interaction profile for TP-427 with classic AEDs was assessed according to the isobolographic transformation method. Although both methods in their names have the same word—“isobolographic”, they differ significantly. In the isobolographic transformation method, the tested drug is administered at a constant and unchanged dose, which by itself does not have a statistically significant effect on seizure excitability threshold in mice [28, 49] (e.g., 5 or 10 mg/kg for TP-427). In the classic isobolographic analysis method, both TPL-16 and classic AED doses increase gradually in a constant ratio of 1:1, so the obtained ED_50 mix_ value refers to a mixture of both compounds, and not only to a classic AED [50, 51]. Additionally, in the classic isobolographic analysis method, all the drugs used increase the seizure excitability threshold in mice, i.e., exert a clearly defined anticonvulsant effect in the MES test in mice. Previous studies carried out for 1,2,4-triazole-3-thione derivatives have shown that some agents, including TP-4 and TP-10 increased the threshold for electrically-evoked convulsions in mice [20, 24]. However, the assessment of their effect on the anticonvulsant effects of classic AEDs was carried out using doses of the investigated substances that by themselves did not significantly affect the threshold for maximal electroshock-induced seizures in mice [49]. Previously, it has been found that TP-10 increased the anticonvulsant effect of valproate and at the same time did not significantly affect the anticonvulsant protective effect of carbamazepine, phenytoin and phenobarbital in the MES test in mice [24]. In turn, TP-4 enhanced the anticonvulsant protective effect of carbamazepine, phenobarbital and valproate, but not phenytoin in the MES test in mice [20].

It should be emphasized that the preclinical assessment of the nature of the interaction of TPL-16 with classic AEDs makes it possible to assess whether TPL-16 could be used as an add-on drug in epilepsy therapy in humans. In the case of an ineffective therapy of epilepsy in humans, the alternative monotherapy, consisting of replacing an ineffective drug with another AED administered alone, is used [5, 52, 53]. However, if three consecutive monotherapy medications are not effective and the patient is still at risk of epileptic seizures, then polytherapy based on the combined administration of at least two AEDs is considered [5, 52–54]. In such a situation, the combination of AEDs is based on rational premises, according to which the molecular mechanisms of AEDs, their safety profile during application, development of tolerance and the possibility of pharmacokinetic interactions are considered [55, 56]. New AEDs, in clinical conditions, are usually combined with classic AEDs with known pharmacological action profiles. Therefore, it is not surprising that preclinical studies first evaluate the effectiveness of a combination of classic AEDs with TPL-16—a potential candidate for a new AED. In addition, during the replacement of one ineffective AED with another drug, a patient is administered for a certain period of time with so-called transient polytherapy, and the interactions that occur then play an important role because they can change the profile of both drugs. This method of clinical therapeutic management was tried to be reproduced in preclinical studies in mice.

To assess the type and nature of interactions between TPL-16 and classic AEDs in the MES test in mice, 2 independent methods were used, which allow determining the strength and type of interaction precisely. The first method was statistical analysis of data using the unpaired Student’s t-test, which allows to clearly indicate whether the existing difference between ED_50 add_ and ED_50 mix_ values ​​is statistically significant at the appropriate level (usually, it is set up at *p* < 0.05). At present, there is no doubt that statistical analysis of results based on the Student’s t-test is the only acceptable method to isobolographically assess the nature of drug-drug interactions [29]. The second method that was also used in this study to classify interaction is the method based on calculating the interaction index, which is the quotient of ED_50 mix_ and ED_50 add_ values [30, 31]​​. For each tested combination of TPL-16 with classic AEDs in the MES test in mice, the interaction index was calculated that ranged from 0.83 to 0.66. In this method, the nature and type of interaction is determined by the value limit of interaction index, and thus, this measure is considered not very precise. If the value of the interaction index is less than 0.7 then the interaction is assumed to be synergistic. The interaction index values ​​between 0.7 and 1.3 describe additive interactions, while antagonism is described for interaction index values ​​above 1.3 [28]. For the combination of TPL-16 and valproate, the interaction index was 0.66 indicating a synergistic interaction, which was also confirmed during statistical analysis by means of the Student’s t-test (*p* < 0.05). In turn, combinations of TPL-16 with carbamazepine, phenytoin and phenobarbital in the MES test in mice showed interaction indices in the range of 0.7–1.3, which clearly classify interactions as additive. The absence of statistical significance between the ED_50 mix_ and ED_50 add_ values for the respective combinations confirmed the existence of additive interactions.

Considering the nature of the interaction of TPL-16 with classic AEDs, it should be emphasized that the most beneficial is the one found between TPL-16 and valproate, showing synergy in the anticonvulsant activity in the MES test in mice. Given the molecular effects of TPL-16, which belongs to the group of 1,2,4-triazole-3-thione, it is assumed that TPL-16 does not bind to benzodiazepine binding sites and has no (direct or allosteric) effect on GABA_A_ receptors and nicotinic acetylcholine receptors built of α7 and α4β2 subunits [14], but its anticonvulsant effect is probably due to its effect on voltage-gated sodium channels [18]. Although this has not been proven directly for TPL-16, other 1,2,4-triazole-3-thione derivatives such as TP-315 block voltage-gated sodium channels [18]. In turn, valproate has many different mechanisms of action, the most important of which are increasing GABA synthesis and release, enhancing GABAergic transmission through enhancing GABA postsynaptic action in specific brain regions [57]. Valproate reduces the release of the beta-hydroxybutyric excitatory amino acid and reduces the neuronal stimulation of NMDA ionotropic receptors for excitatory amino acids [58]. In addition, valproate blocks voltage-gated sodium channels, blocks T-type low voltage calcium channels, modulates dopaminergic and serotoninergic transmission in the brain [57, 58]. There is no doubt that multimodal effects of valproate in combination with TPL-16 contribute to the synergistic inhibition of MES-induced seizures in mice.

The next stage of the experimental study was to assess whether combinations of TPL-16 and classic AEDs, at doses corresponding to individual ED_50 mix_ values ​​for a fixed dose ratio of 1:1 from the MES test, did not cause adverse effects in 2 behavioral tests in mice—chimney and grip-strength tests. It is generally accepted that the chimney test in mice is used to assess potential adverse drug effects on movement and coordination in rodents [32]. The disturbance of motor coordination is manifested in the chimney test by the inability of the animal to go out of the back from a vertically positioned cylinder 30 cm long in 1 min Mice with impaired motor coordination cannot climb backwards up the rough inner surface of the cylinder and slide downwards, and these impairments are most often due to the disruptive effect of drugs on neurotransmission in the cerebellum and brain structures responsible for coordinating muscle movement on the right and left side of the animal’s body [32]. In turn, the grip-strength test allows assessing the side effects of drug combinations that may impair skeletal muscle strength in rodents [33]. The weakening of skeletal muscle strength in mice is usually due to the disruptive effect of drugs on neuromuscular transmission, which may result in muscle weakness and even muscle flaccidity [33]. Both, grip-strength and chimney tests showed no effect of the combinations of TPL-16 with carbamazepine, phenytoin, phenobarbital and valproate on the central nervous system in rodents. It should be stressed that the combinations of TPL-16 with classic AEDs were not tested at doses producing acute neurotoxic effects, but only at doses offering the antiseizure effects from the MES test. Thus, we found that in the chimney and grip-strength tests the combinations at doses offering protection from MES-induced seizures, exerted no acute adverse effects in mice. This is the reason that the protective index was calculated only for TPL-16 alone, but not for the combinations of TPL-16 with classic AEDs.

At the last stage of experimental studies, the brain concentrations of the classic AEDs administered separately and in combination with TPL-16 were determined in constant dose ratios derived from the MES test in mice. These studies aimed to determine whether TPL-16 significantly changes the concentration of classic AEDs in mice. The obtained results clearly indicated the lack of significance between drug concentrations in the control group receiving the AED alone and in the group receiving the combination of TPL-16 with the AED. It should be emphasized that during the experiments, it was planned to assess the concentration of classic AEDs in mouse brain homogenates, i.e., at the site of drug action, not in animals’ serum, as it was preferentially done in experimental studies at the end of the twentieth century. Previous studies have experimentally shown that concentrations of AEDs determined by polarized fluorescence and HPLC may differ between serum and brain homogenates. An evident example of different concentrations of AEDs in serum and brain homogenates in mice was the assessment of influence of 2-phosphonomethylpentanedioic acid (2-PMPA—a glutamate carboxypeptidase II inhibitor) on valproate levels. It has been experimentally shown that 2-PMPA significantly increased the brain concentration of valproate by 37% (*p* < 0.001), while the concentration of valproate in the serum decreased only by 5% [59]. Finally, it was experimentally demonstrated that the 2-PMPA enhanced the protective effect of valproate in the MES test in mice, and the observed interaction was pharmacokinetic in nature associated with an increase in valproate concentration in brain homogenates, with no significant changes in serum drug concentrations in mice [59].

Noteworthy, in this study TPL-16 did not affect concentrations of classic AEDs and, thus the observed interactions between classic AEDs and TPL-16 had probably a pharmacodynamic background. In turn, TP-427 significantly increased the concentration of valproate in brain homogenates in mice, whereby the observed synergistic interaction between TP-427 and valproate in the MES test in mice had a pharmacokinetic component [22]. On the other hand, TPF-34 administered in combination with valproate did not significantly change valproate concentrations in mouse brain homogenates, thus the observed additive interaction in the MES test in mice was pharmacodynamic. On the other hand, the combination of TP-10 with valproate showed a 52% significant increase in valproate concentration (*p* < 0.001), whereby the observed combination in the MES test in mice had a pharmacokinetic component [24]. A similar type of interaction was observed for TP-4 in combination with valproate, for which a significant increase in valproate concentration by 40% (*p* < 0.001) in brain homogenates in mice was demonstrated [20]. Given the nature of the interaction between the different 1,2,4-triazole-3-thione derivatives and valproate, it should be noted that TPL-16 is similar to TPF-34, since in both cases the nature of the interaction was pharmacodynamic. In turn, TP-4, TP-10 and TP-427 significantly increased the brain concentrations of valproate in mice, whereby the interactions observed in the MES test in mice had a pharmacokinetic component. It should be emphasized here that the pharmacokinetic verification of drug concentrations in brain homogenates in mice was carried out in this study only for classic AEDs, but not for TPL-16 and its potentially active metabolites. Of note, in this study we did not measure total brain TPL-16 concentrations, but only concentrations of classic AEDs. The lack of such measurement resulted from difficulties in the detection of TPL-16 in animals. Although concentrations of classic AEDs were measured with FPIA technique, the detection of TPL-16 needs more advanced pharmacokinetic studies related with evaluation of time to peak effect and half-life for TPL-16, as well as, the analysis of the active compounds, resulting from metabolic transformation of TPL-16. At present, the analysis of basic pharmacokinetic parameters for TPL-16, when used either alone or in combination with classic AEDs, needs to be performed in mice. Previously published results revealed that estimation and measurement of TP-315 (another 1,2,4-triazole-3-thione derivative) in the mouse brain tissue required a special technique based on the reversed-phase high performance liquid chromatography (RP-HPLC) with photo-diode array detection (DAD) [19]. Because of structural similarities between TP-315 and TPL-16, the same methodological approach is expectedly required to detect TPL-16 in the brain tissue of the experimental animals. It should be stated that the lack of estimation of TPL-16 concentrations in the animals’ brain tissue is the main limitation in this study.

Considering all aspects of the study mentioned above, the combination of TPL-16 and valproate is beneficial and recommendable for further research study because the synergistic drug interaction in anticonvulsant protection in the MES test in mice is not accompanied by any pharmacokinetic interaction and this combination does not cause any acute undesirable effects in behavioral tests in animals. Other combinations of TPL-16 with carbamazepine, phenytoin and phenobarbital, although they show additive interaction with respect to the anticonvulsant protection from tonic-clonic seizures in mice, they may also be beneficial in some patients, but this requires confirmation in further experimental studies using other seizure models.

## Data Availability

All data are presented in tabular and graphical forms. Additionally, the data could be available upon request.

## Abbreviations

AEDs: Antiepileptic drugs
MES: Maximal electroshock-induced seizure model
TPL-16: 4-butyl-5-[(4-chloro-2-methylphenoxy)methyl]-2,4-dihydro-3*H*-1,2,4-triazole-3-thione

## Code Availability

G*Power, RRID:SCR_013726 GraphPad Prism, RRID:SCR_002798.
